# Beyond Verb Meaning: Experimental Evidence for Incremental Processing of Semantic Roles and Event Structure

**DOI:** 10.3389/fpsyg.2017.01806

**Published:** 2017-10-30

**Authors:** Markus Philipp, Tim Graf, Franziska Kretzschmar, Beatrice Primus

**Affiliations:** Institute of German Language and Literature I, University of Cologne, Cologne, Germany

**Keywords:** agentivity, animacy, semantic role, locative vs. goal adverbial, event structure, motion verb, eventrelated brain potentials (ERP), N400

## Abstract

We present an event-related potentials (ERP) study that addresses the question of how pieces of information pertaining to semantic roles and event structure interact with each other and with the verb’s meaning. Specifically, our study investigates German verb-final clauses with verbs of motion such as *fliegen* ‘fly’ and *schweben* ‘float, hover,’ which are indeterminate with respect to agentivity and event structure. Agentivity was tested by manipulating the animacy of the subject noun phrase and event structure by selecting a goal adverbial, which makes the event telic, or a locative adverbial, which leads to an atelic reading. On the clause-initial subject, inanimates evoked an N400 effect vis-à-vis animates. On the adverbial phrase in the atelic (locative) condition, inanimates showed an N400 in comparison to animates. The telic (goal) condition exhibited a similar amplitude like the inanimate-atelic condition. Finally, at the verbal lexeme, the inanimate condition elicited an N400 effect against the animate condition in the telic (goal) contexts. In the atelic (locative) condition, items with animates evoked an N400 effect compared to inanimates. The combined set of findings suggest that clause-initial animacy is not sufficient for agent identification in German, which seems to be completed only at the verbal lexeme in our experiment. Here non-agents (inanimates) changing their location in a goal-directed way and agents (animates) lacking this property are dispreferred and this challenges the assumption that change of (locational) state is generally a defining characteristic of the patient role. Besides this main finding that sheds new light on role prototypicality, our data seem to indicate effects that, in our view, are related to complexity, i.e., minimality. Inanimate subjects or goal arguments increase processing costs since they have role or event structure restrictions that animate subjects or locative modifiers lack.

## Introduction

An essential part of sentence comprehension is distinguishing the sentential arguments and interpreting their respective semantic roles. Semantic roles (also thematic relations or roles) capture certain generalizations about the participation of entities in eventualities denoted by linguistic expressions regarding such issues as who did it, whom it happened to and what got changed. A widely held view in linguistics is that “they label relations of arguments to predicators and therefore have no existence independent of predicators.” (Rappaport and Levin, 1988, p. 17; repeated in Davis, 2011, p. 401). Thus, for example, the participant named *John* is a volitional instigator, i.e., an agent, in relation to *bake*, e.g., *John baked the cake*, and a perceiver in relation to *see*, e.g., *John saw the cake*. According to this assumption, the lexical meaning of the verb determines the semantic roles of its arguments. The lexical meaning of the verb also determines event structure (i.e., aspect). The verb *see* in *John saw the cake* refers to a state, the verb *bake* in *John baked the cake* to an accomplishment, i.e., a telic event (Vendler, 1967). The main debate centers around the question whether the verb’s lexical meaning is the only factor in determining event structure and semantic roles or whether there are additional ingredients, including the meaning of the arguments themselves (e.g., animate vs. inanimate). These additional factors have gained importance, as there is growing consensus that verb meanings are often indeterminate with respect to their role selection and event structure. A pertinent case are verbs of motion in German (see in (1) below), which are indeterminate with respect to event structure (Lukassek et al., 2016) and agentivity and which are in the focus of our investigation. In psycholinguistics, it is widely assumed that semantic role information is processed incrementally even before the verb lexeme is encountered, a good testing case being languages with verb-final sentences (e.g., Kamide et al., 2003a for Japanese and Kamide et al., 2003b; Kretzschmar et al., 2012, for German). But the question of how semantic role and event structure information is processed incrementally before the verb is encountered in German has not been investigated experimentally before.

This article presents an event-related potentials (ERP) study on verb-final clauses in German, which allow us to investigate the incremental interpretation of each major constituent before the verb lexeme in the clause is encountered. The ERP method reflects the discrete time course dynamics of language processing relative to the onset of a stimulus; by that it is particularly well-suited to investigate whether and how information presented prior to the verb lexeme is integrated step-by-step into the meaning of the clause. Specifically, we investigate the incremental processing of an animate or inanimate subject argument (*der Gleitschirmflieger* ‘the paraglider’ vs. *das Ahornblatt* ‘the maple leaf’) followed by a locative or goal phrase (*über dem Fluss* ‘above the river’ vs. *auf den Acker* ‘to the ground’) and a semantically indeterminate verb of motion such as *schweben* ‘float,’ which may select the auxiliary *sein* ‘be’ or *haben* ‘have.’ Cp. (1):

(1) *Dass der Gleitschirmflieger / das Ahornblatt über dem Fluss / auf den Acker*
That the paraglider / the maple leaf above the river / to the ground
*geschwebt ist/hat, faszinierte den Fußgänger*.
floated is/has, fascinated the pedestrian.
‘That the paraglider/the maple leaf floated above the river/ to the ground fascinated the tourist.’

Let us introduce the animacy distinction, which is manifest in the initial noun phrase. Importantly for our study, animacy is central to the interpretation of the more complex notion of agentivity (e.g., Rakison and Poulin-Dubois, 2001; Dahl, 2008). We take a broad view on the agent notion by including in addition to volitional instigators also self-propelled movers, experiencers of emotions and perceivers in perception events into a generalized notion of agentivity (cf. proto-agent in Dowty, 1991; Primus, 1999, 2012). These agentive roles imply animacy of the participant and as a consequence, an animate referent indicates that it is well-suited for the proto-agent role by virtue of the fact that it is sentient, capable of self-initiated motion and volitional action. Correspondingly, it is difficult to classify inanimates incapable of sentience, volition and self-initiated motion as proto-agents (Muralikrishnan, 2011; Primus, 2012).

In our test items, the initial noun phrase is either unambiguously a nominative (e.g., *der* + noun masc, sg, nom) or indeterminate non-oblique (e.g., *das* + noun neutr, sg, nom or acc; *die* + noun fem, sg, nom or acc). The unambiguously nominative noun phrases are interpreted as the subject of the sentence according to their case; the case-indeterminate noun phrases are preferentially interpreted as the subject due to the robust subject-first processing strategy (e.g., Schriefers et al., 1995; Kretzschmar et al., 2012, for German). There are several ERP studies examining animacy effects at the subject noun phrase. In their ERP study of English, Weckerly and Kutas (1999) report a central negativity for initial inanimate arguments against animate counterparts between approximately 200 and 500 ms, classified as N400. The animacy effect was observed for the position of a sentence initial head noun phrase as well as for the subject noun phrase of a subsequent relative clause, as shown in (2a, b). Here and in (4a, b) noun phrases that elicited an N400 effect (i.e., increased amplitude relative to another condition) are shaded.

(2) (a) *The 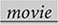 that the novelist praised inspired the director....*
(b) *The 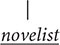 that the 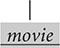 inspired praised the director...*

Likewise, Muralikrishnan (2011) observed an N400 for sentence-initial inanimate nominative subjects as opposed to animate (nominative and dative) subjects in transitive clauses in Tamil.

These observations for English and Tamil are contrary to findings from transitive clauses in German, where previous ERP studies reveal no differences between inanimate and animate initial arguments (see for an overview Bornkessel-Schlesewsky and Schlesewsky, 2009a). Likewise, ERP data from transitive sentences in Mandarin Chinese (Philipp et al., 2008) did not reveal animacy effects at the initial noun phrase.

In sum, pertinent ERP research reports different results, which lead to different predictions for our study. The above-mentioned studies on German and Chinese lead to prediction (3a), the results for English and Tamil to prediction (3b). Since the transitive clause structures tested previously for German, Chinese, English, and Tamil differ from the intransitive clause structure tested in the current experiment, we consider both predictions to be viable options for our data.

(3) (a) The initial noun phrase will evoke no animacy-related processing effects.
(b) Inanimate initial noun phrases will elicit an increased N400 against animate ones.

Let us also discuss the explanations offered for the lack or presence of animacy effects on the initial argument in previous research. Bornkessel-Schlesewsky and Schlesewsky (2009a) explain the absence of animacy effects on initial arguments in German by assuming that animacy only plays a secondary role in the processing of the initial noun phrase in German. In their opinion, animacy becomes important only as a relational feature when two or more arguments must be integrated and interpreted relative to one another as proto-agent and proto-patient. What is difficult to process in German is, in their view, a constellation in which an inanimate and hence non-prototypical agent follows an animate patient. This assumption is based on the results of Roehm et al. (2004) and Ott (2004, see Bornkessel-Schlesewsky and Schlesewsky, 2009a, p. 31). Roehm et al. (2004) report that inanimate nominative arguments following an initial animate accusative elicit an N400 effect against animate nominative arguments. Cf. (4a, b):

(4) (a) *welchen Förster 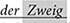 streifte*
which ranger (ACC) the branch (NOM) brushed
(b) *welchen Angler* 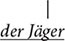 *lobte*
which angler (ACC) the hunter (NOM) praised

Roehm et al. (2004) did not include initial nominatives in their study, but in an experiment using similar stimuli no comparable effect was observed for initial nominatives referring to inanimates (Ott, 2004, see Bornkessel-Schlesewsky and Schlesewsky, 2009a, p. 31). Likewise, ERP data from transitive sentences in Mandarin Chinese (Philipp et al., 2008) reveal animacy effects at the second argument but not at the initial noun phrase. As in German [see the overt nominative marking of *der* ‘the-NOM’ in (4a,b)], the second argument was unambiguously a proto-agent.

The above-mentioned studies on German tested transitive constructions with two preverbal nominal arguments. As the items of these studies employed a similar constituent order even in filler sentences, it seems reasonable to consider that the item structure itself may have shaped a particular incremental expectation. Due to the transitive stimulus structure, participants may have always expected a second argument, thereby being used to interpret animacy as a relational feature between two arguments. This might have postponed the evaluation of animacy from the first noun phrase to the second. In our study, the item structure, which was also uniform across all conditions including fillers, might have played a role as well. The initial noun phrase in our design is expected to be the single nominal argument of the clause so that this is the only nominal position where animacy could be evaluated. This is the reason why we do not consider prediction (3a) to be the only option for German.

Let us turn to possible explanations for an animacy effect, as predicted by (3b). An interesting proposal is suggested by the assumption of Weckerly and Kutas (1999, p. 566) that animate subjects are barely restricted in terms of verb types (e.g., *hit, remain)*. In our view, this entails that they are also barely restricted in terms of semantic roles: relative to action verbs like *hit* and *work*, the subject is a volitional agent; relative to stative verbs such as *remain* and *seem* it is a theme. Other possible roles are self-propelled mover (e.g., *roll*), experiencer (e.g., *feel cold*), perceiver (e.g., *see*) and patient (*be hit*). This means, in our view, that animate subjects are indeterminate (or underspecified) with respect to their semantic role. By contrast, it is more difficult or impossible to interpret inanimates as volitional agents, experiencers, perceivers or self-propelled movers. Thus, when encountering an animate subject, the processing system may keep role identification to a minimum. By contrast, when encountering an inanimate subject, it is confronted with increased processing costs by eliminating the above-mentioned agent properties as an option. Likewise, the results of Paczynski and Kuperberg (2009) suggest that animacy is insufficient for role identification in English. They found an N400 on an initial inanimate subject in both active and passive clauses and no interaction between voice and animacy on the verb. If the animate-inanimate opposition would have been interpreted as an agent–patient contrast, inanimate patient subjects should have had a processing advantage against animate subjects in the passive, but this was not the case. This type of explanation can be subsumed under the more general minimality processing principle (e.g., Hawkins, 2004; Bornkessel et al., 2004; Bornkessel-Schlesewsky and Schlesewsky, 2009b): During online comprehension semantic (and syntactic) specifications are kept to a minimum until more information is present either in the utterance itself or the context.

Muralikrishnan (2011, p. 108) offers a different explanation for the animacy effects on initial nominative arguments in Tamil. Muralikrishnan assumes that as soon as an animate nominative argument is encountered during online sentence processing, it is assigned a prototypical agent role. Inanimate nominative arguments are more difficult to process because they cannot be interpreted as a prototypical agent. Since non-prototypical role constellations are known to elicit an N400 (e.g., Philipp et al., 2008; Bornkessel-Schlesewsky and Schlesewsky, 2009a; Nieuwland et al., 2013) this type of explanation also predicts an N400 effect for inanimate nominative arguments [see (3b) above].

The minimality explanation we derived from Weckerly and Kutas (1999) and the prototypicality-based explanation offered by Muralikrishnan (2011) both predict additional processing costs in form of an increased N400 for the inanimate condition. Therefore, it is not possible to disentangle the two types of explanation if one takes only animacy of the subject into consideration. A relation between animacy and a second factor is needed to dissociate these explanations, as already suggested by the findings of Paczynski and Kuperberg (2009), who investigated the interaction between animacy and voice. We expect that in our study the two explanations can be teased apart by taking telicity as an additional factor into consideration. This factor is varied at the adverbial phrase, to which we turn now.

The second pertinent variation in our experiment is the locative vs. goal adverbial phrase. The prototypical adverbial is optional and corresponds syntactically to an adjunct, acting semantically as a modifier (Maienborn and Schäfer, 2011). Locative adverbials belong to this type in general. There are only very few verbs selecting a locative argument, e.g., *New York lies ^∗^(on the Hudson river bank*). By contrast, the prototypical function of a goal phrase is that of an oblique argument (Maienborn, 1994). Goal phrases occur in complex events which include a change-of-location component. Such events are classified as telic. In many cases the goal phrase itself adds this meaning component (e.g., *er tanzte in den Saal* ‘he danced into the hall,’ see Maienborn, 1994). By contrast, a locative phrase does not increase the complexity of the event structure. If the event structure of the verb meaning is indeterminate, as in our experiment, the event structure of an item with a locative modifier is indeterminate too, since the locative modifier adds no change of state component. Due to the fact that a goal adverbial adds complexity to the clause in terms of number of argument slots and event structure, we predict that it will show increased processing costs vis-à-vis a locative adverbial. Our prediction is backed by processing considerations. The assumption that the processing system endeavors to minimize linguistic (e.g., syntactic and semantic) specifications during online comprehension has a long standing-tradition in psycholinguistics (e.g., Hawkins, 2004; Bornkessel et al., 2004; Bornkessel-Schlesewsky and Schlesewsky, 2009b). Since a shift from a minimal one-argument template to a two-argument template has been shown to elicit an N400 effect (Bornkessel-Schlesewsky and Schlesewsky, 2009b), we expect to find this effect at the goal argument position in our study. Cp. (5):

(5) The goal argument will require additional processing costs reflected in an increased N400 against the locative non-argument phrase.

However, this prediction is tentative since Bornkessel-Schlesewsky and Schlesewsky (2009b) have found an N400 on a second nominal argument, while in our study, the complexity increase is due to a second oblique goal argument.

Our tentative prediction (5) seems to contradict previous research on the atelic-telic distinction in English (Malaia et al., 2009, 2012; Malaia and Newman, 2015). In these studies, telicity has been shown to facilitate integration of an internal (non-agent) argument and syntactic reanalysis in reduced object relative clauses (e.g., *the actress awakened/worshipped by the writer left in a hurry*). In the atelic condition (*the actress worshipped*) the semantic role of the initial argument and the sentence structure has to be reanalyzed at the point of disambiguation (*by* for participants with high comprehension proficiency): the initial agent role assignment has to be revised. The telic condition (*the actress awakened*) does not necessitate such reanalysis. Malaia and colleagues report ERP modulations in time windows before the N400 (i.e., in N100 and P200 time range), and linked these effects to semantic role reanalysis and change of argument templates. However, our stimulus material differs from that of the above-mentioned studies in several relevant ways, which in turn renders it difficult to derive predictions for our experiment based on these prior studies. Firstly, the intransitive motion verbs in our study are indeterminate with respect to telicity (see Lukassek et al., 2016 for behavioral experimental evidence) and the verbs are presented after the goal or locative phrase. By contrast, the verbs used in the above-mentioned previous studies for English were unambiguously either telic or atelic and were presented before the point of disambiguation. Secondly, the telic-atelic opposition was tied to lack vs. need of reanalysis (i.e., the effects occurred only in temporarily ambiguous relative clauses that required reanalysis in Malaia et al., 2012), whereas the current stimuli do not require reanalysis. Finally, Malaia and colleagues found ERP effects in response to single function words (preposition *by* or determiner *the*), which are known to generally elicit smaller N400 components than content words (cf. Neville et al., 1992; Nobre and McCarthy, 1994) and therefore make it less likely to find effects in mean N400 amplitude. In contrast, we measured ERPs for entire goal/locative phrases and on the verbal participle, thereby including content words as eliciting events.

There might be an interaction between the animacy of the subject noun phrase and the telicity value indicated by the locative vs. goal phrase. This interaction has been discussed in previous research in connection with the semantic classification of verbs. Therefore, we will discuss the verbal participle first and return later to a potential animacy–telicity interaction at the adverbial phrase.

The third and last constituent of interest in our experiment is the verbal past participle. At this point, three pieces of information are assembled: animacy information, the event structure inferred from the semantics of the adverbial phrase, and the verb meaning referring to a process of motion. The interaction between semantic roles and event structure in dynamic (e.g., motion) events has been discussed extensively in the linguistic literature. In Dowty’s (1991) work, for instance, moving gradually toward a definite goal is a definite change of state, which is an important property in his cluster definition of proto-patient [see Dowty’s (1991, p. 567–572)] elaborations on ‘incremental theme,’ see also Ackerman and Moore, 1999). This analysis is in conformity with the common assumption that verbs of motion referring to a change of location belong to the class of ‘unaccusative,’ verbs that select a patient or a theme (Zaenen, 1993, p. 142). The predictions derived from this line of research are as follows. The semantic role of an animate entity undergoing a definite change of location has inconsistent role properties. As an animate argument of a motion verb it is preferentially interpreted as an agent, by going through a definite change of location it is a patient. Correspondingly, an inanimate entity lacking the crucial patient property of a definite change of state is expected to be a less prototypical patient and to cause higher processing costs than an inanimate entity undergoing a definite change of state. Differences regarding role prototypicality have been shown to modulate the N400 component in previous ERP research (e.g., Philipp et al., 2008; Bornkessel-Schlesewsky and Schlesewsky, 2009a; Nieuwland et al., 2013). In conjunction with the above-mentioned assumption of an inverse correlation between agentivity and telicity in the linguistic literature, this line of ERP research leads to the prediction formulated in (6).

(6) TelAgInverse: Animates in the telic (goal) condition and inanimates in the atelic (locative) condition require additional processing costs reflected in an increased N400 amplitude against animates in the atelic (locative) condition and inanimates in the telic (goal) condition.

A different picture emerges from research on ontogenetic and phylogenetic language development. Many experimental studies and meta-analyses of such studies concur in the claim that goal-directed behavior characterizes agents, among other properties such as autonomous, i.e., self-propelled, movement. For Rakison and Poulin-Dubois (2001), for example, a purpose of action (goal-directed vs. without aim) is one of the seven characteristic properties related to causality that distinguish animates from inanimates already in infant cognition (cp. also Carpenter et al., 2005; Spelke and Kinzler, 2007; Carey, 2009). In this view, a participant that changes his/her location in a goal-directed way independently of another participant is a more prototypical agent than a participant that moves aimlessly or whose movement is caused by another participant. The predictions of this line of research – formulated in semantic terms – are as follows. An inanimate entity involved in a definite (goal-oriented) change of location has inconsistent role properties. As an inanimate entity it is preferentially interpreted as a patient, its goal-oriented behavior would qualify it as an agent. Correspondingly, an animate referent moving aimlessly is a non-prototypical agent lacking the property of goal-directedness. Consequently, this line of research leads to predictions that are contrary to the ones derived from the linguistic analysis mentioned above. See (7):

(7) TelAgHarmonic: Animates in the atelic (locative) condition and inanimates in the telic (goal) condition require additional processing costs reflected in an increased N400 amplitude against animates in the telic (goal) condition and inanimates in the atelic (locative) condition.

Let us return to the adverbial phrase and discuss the interaction between the animacy of the subject noun phrase and the telicity value indicated by the locative vs. goal phrase. The predictions regarding this interaction depend on several premises. Let us assume that we find an animacy effect on the subject, as predicted by (3b). In this case, there are two competing explanations for this effect, as mentioned earlier. Muralikrishnan (2011), for example, assumes that animate subjects are preferentially interpreted as agents even before the verb is encountered. Under this premise, the predictions we formulated for the verbal participle in (6) and (7) are already applicable at the position of the adverbial phrase, since agent identification is already completed at the subject position and the telicity value is determined by the locative vs. goal phrase before the verb is encountered. TelAgInverse in (6) predicts an increased N400 for the adverbial phrase in the animate-telic and inanimate-atelic conditions due to early agent identification and the assumption that agentivity and telicity correlate negatively. By contrast, TelAgHarmonic in (7) predicts an increased N400 for the adverbial phrase in the animate-atelic and inanimate-telic conditions due to early agent identification and the hypothesis that goal-directedness is an agent property.

But there is also the possibility that during online processing animate subjects are left underspecified in terms of their semantic role (e.g., Paczynski and Kuperberg, 2009). If role identification occurs only later, at the verb position, TelAgInverse and TelAgHarmonic are irrelevant for the processing of the adverbial phrase. The same holds under the premise that there is no animacy effect, as predicted by (3a). In both cases, we expect only a minimality-driven increased N400 in the telic (goal) conditions [see (5) above] and possibly also in the inanimate conditions, i.e., animate-telic, inanimate-telic and inanimate-atelic will differ from animate-atelic.

## Experiment

### Subjects

Twenty-nine monolingual native-speakers of German (22 females, mean age 24.8, undergraduate students at the University of Cologne) participated in this experiment after giving written informed consent. None of them reported reading or speech disorders nor any neurological impairments. All participants were right-handed (assessed by an adapted German version of the Edinburgh handedness questionnaire, Oldfield, 1971).

This study was carried out by the Experimental Linguistics Lab at the University of Cologne (XLinC) in accordance with the national and institutional recommendations adopted by XLinC which the authors are members of. XLinC received an ethics approval from the Ethics Committee of the German Linguistic Society (Deutsche Gesellschaft für Sprachwissenschaft, [DGfS]^1^) for conducting non-invasive behavioral, eye tracking, and EEG studies with healthy adults (age between 18 and 65) that is in full conformity with the guidelines of the Deutsche Forschungsgemeinschaft (German Research Council, DFG) for non-invasive studies, such as EEG^2^. All subjects gave written informed consent in accordance with the Declaration of Helsinki.

### Materials and Design

#### Stimulus Structure

As the time course of sentence processing is of particular interest for us, the order in which the stimuli are presented is crucial (usually, as in our experiment, a word or a phrase in a rapid serial visual presentation (RSVP) method). The incremental processing flow not only involves an evaluation of the current input with respect to various linguistic information types (e.g., word order, morphological and lexical-semantic information), it also involves evaluation of these types of information with respect to preceding information units (e.g., associative-semantic and morpho-syntactic interpretation, cf. e.g., Marslen-Wilson, 1973; Crocker, 1994; Stabler, 1994) as well as computation of particular and general predictions with regard to potentially upcoming input and estimated interpretations (cf. Stabler, 1994; Kutas and Federmeier, 2000; Hagoort et al., 2004). Our main research question is to investigate the contribution of the verb’s co-constituents to the interpretation of semantic roles and event structure and to explore how these pieces of information are assembled step-by-step with respect to each other and with the verb’s meaning. To this end, a stimulus order is required where the verb’s co-constituents precede the verb lexeme in basic order. Basic (i.e., unmarked) order of the major constituents in a clause is important since it is unaffected by possibly intervening discourse-related factors such as information structure (e.g., Lenerz, 1977). For the same reason, the structure containing the critical items should not be preceded by additional material as far as possible. The structure shown in (1) and in **Table 1** fulfills these criteria and has been selected for our experiment. The only element preceding our critical items is a clause-initial complementizer devoid of lexical meaning.

**Table 1:** Examples for all eight conditions for *schweben* ‘float, hover.’

Condition	Animacy	Telicity		Auxiliary
Animate-atelic (loc), *sein*/*haben*	*Dass der Gleitschirmflieger letzten Mittwoch* That the paraglider last Wednesday	*über dem Fluss* above the river		*ist*, / *hat*, is / has
Animate-telic (goal), *sein*/*haben*		*auf den Acker*		*ist*, / *hat*, is / has
		to the ground		
			geschwebt	
Inanimate-atelic (loc), *sein*/*haben*	*Dass das Ahornblatt letzten Mittwoch* That the maple leaf last Wednesday	*über dem Fluss* above the river	floated	*ist*, / *hat*, is / has
Inanimate-telic (goal), *sein*/*haben*		*auf den Acker*		*ist*, / *hat*, is / has
		to the ground		

Main clause	*faszinierte den Fußgänger*
	fascinated the pedestrian

*Abbr.: loc = locative*.

In order to draw participants’ attention away from the critical material – i.e., subject phrase, adverbial phrase and verb lexeme –, we introduced the two auxiliaries *haben* vs. *sein* as a further manipulation, as shown in (1) above. Participants’ task in our ERP experiment was to judge sentence acceptability [see Procedure and Behavioral Data (Acceptability Judgments)]. The auxiliary manipulation is expected to yield strong acceptability differences for the following reason. As mentioned above, the verbs of motion under study are indeterminate with respect to animacy and telicity. This means that they can be used with both animate and inanimate subjects and with both goal and locative phrases. By contrast, auxiliary selection for these verbs is strongly biased in favor of *sein*. Taking this into consideration, we predict that telic clauses with *haben* will be predominantly unacceptable leading to a sharp acceptability contrast vis-à-vis telic clauses with *sein*. This sharp acceptability contrast is in our view suitable to draw participants’ task-related attention away from the critical items. The additional advantage is that in our stimuli the auxiliary always follows the participial verbal lexeme, thereby excluding task-related ERP effects at or before the verb lexeme position. Finally, by using the perfect tense formed of verb participle and inflected auxiliary, we are able to separate lexical-semantic verb information from morpho-syntactic agreement information. Hence, we are able to use the variability of the critical verbs with respect to auxiliary selection as an experimental condition that serves both as a highly salient distractor (task-induced focusing on auxiliary selection) and as a task-related control condition.

#### Materials

We investigate the interplay of agentivity and telicity in a fully crossed and balanced 2 × 2 design by orthogonally manipulating these factors, i.e., agentivity is varied via the factor ANIMACY (animate vs. inanimate subject noun phrase) and the factor TELICITY is changed by using either a goal or locative phrase specifying a telic or atelic reading, as described above (see **Table 1** for examples). Therefore, all verbs used in this experiment should be compatible with an animate or inanimate subject and a goal or locative phrase. Hence, verbs denoting motional processes are a suitable test case. The amount of verbs in German that fulfill all requirements is rather limited. Thus, our material is restricted to six motion verbs that are intransitive, i.e., lack a second nominal argument, or have a predominant intransitive reading in German. The verbs are *fliegen* ‘fly,’ *rollen* ‘roll,’ *schweben* ‘float, hover,’ *schlingern* ‘swerve, lurch,’ *schwimmen* ‘swim,’ *wirbeln* ‘swirl, whirl.’ All verbs can take animate (e.g., *Gleitschirmflieger* ‘paraglider’) or inanimate referents (e.g., *Ahornblatt* ‘maple leaf’) as grammatical subject. With these verbs, telicity can be controlled systematically by adding an adverbial phrase referring to a location (e.g., *über dem Fluss* ‘above the river’ = atelic) or a goal (e.g., *auf den Acker* ‘to the ground’ = telic). A complete set of critical conditions for the verb *schweben* ‘float, hover’ is illustrated in **Table 1**.

We created a total of 480 critical items. For this we paired each of the six verbs with 20 different and contextually plausible noun phrases, 10 denoting animate referents, the other 10 denoting inanimate ones. These nouns were taken from a set of animate and inanimate nouns that did not differ in lexical frequency [unpaired *t*-test: *t*(100) = -0.065, *p* = 0.948; Leipzig Wortschatz frequency corpus^3^. Note that for one word (*Zeppelinfahrer* ‘zeppelin driver’) there was no corpus count]. All nouns denote humans or concrete physical objects.

Always two of those pairs (an animate and an inanimate) were combined with the same contextually appropriate goal or locative phrase. Up to this point, these steps yield 240 critical items. We varied the combinations of lexical nouns and adverbial phrases as often as possible to avoid repetitions as far as possible. Finally, these items were all paired with the two auxiliaries doubling their number to 480. Additionally, we inserted a temporal adverbial between the noun phrase and the locative or goal adverbial in all conditions to exclude an attributive reading of the locative adverbial, i.e., to avoid possible sentence continuations like these: *Dass der Gleitschirmflieger über dem Fluss einen roten Overall trägt* ‘that the paraglider above the river wears a red jumpsuit’ (see **Table 1** above). The resulting 480 items were then equally distributed across four experimental lists according to the Latin Square-design yielding 30 instantiations per list per condition.

In addition to the 120 critical stimuli per list, we constructed 120 filler items that entered each list using six lexically telic intransitive verbs (e.g., *entkommen* ‘escape’) as well as six lexically atelic intransitive verbs (e.g., *arbeiten* ‘work’) in a balanced combination with different animate and inanimate noun phrases. Sentence structure was kept identical to the critical conditions by using subordinate clauses that precede their main clause. Fillers were balanced: about 50% were grammatical and plausible, about 50% were implausible or ungrammatical (ungrammatical auxiliary). A complete list of critical and filler stimuli is provided in the Supplementary Materials.

Finally, we would like to explain why we did not include an additional overt baseline/control condition. We compared binary choices on three constituents. A subject is either animate or inanimate, a motion event either has a goal or it has no goal and an intransitive motion verb selects one of the two auxiliaries BE or HAVE; in all three instances, there is no third option that might serve as a control condition.

### Data Acquisition and Analysis

#### Procedure

The experimental sessions were conducted in a dimly lit, sound attenuated room. Participants were seated approximately 1.2 m in front of a 23 inch wide screen. The stimuli were presented visually in a segment-by-segment manner (RSVP, 200 ms inter-stimulus interval, ISI) on a computer screen. Single words such as the participial verb lexeme were presented for 400 ms, noun phrases and adverbial phrases were each presented together as one string for 500 ms. Each trial began with an asterisk presented for 1000 ms in the center of the screen. After the presentation of the final word in a sentence, a question mark (ISI 500) appeared on the screen for the first task. Participants were instructed to respond via a button press (left and right, positive response was balanced over participants) within an interval of 2000 ms to judge the current sentence for acceptability (binary forced-choice task).

Subsequent to the first task, a recognition task was applied to control for attentiveness by presenting a single word or a phrase on the screen. Participants had to decide whether the presented item on the screen was part of the sentence they had just read or not (maximum response time 3000 ms). *YES* and *NO* responses (50% each) were balanced for left and right hand across participants. The next trial started after an inter-trial interval of 1000 ms.

Participants completed a short practice session of ten items that were structurally identical to but not part of the experimental set before starting with the presentation of 240 trials. After each block of 40 sentences, participants took a short break (about 2 min). Including electrode preparation, an experimental session lasted approximately 2.5 h.

#### EEG Recording, Preprocessing and Data Analysis

The EEG was recorded from 24 Ag/AgCl scalp electrodes placed on an elastic cap (Easycap, Herrsching-Breitbrunn, Germany) following the international 10–20 system (impedances < 5 kOhm, 500 Hz sampling rate, BrainAmp amplifier, Brain Products, Gülching, Germany). Data were re-referenced to the linked mastoids offline and then filtered offline (bandpass.3 – 20.0 Hz). Electrooculogram was recorded from four electrodes at the outer canthi (left, right) and above and below the left eye. EEG data were analyzed using EEProbe software (ANT Enschede, The Netherlands). The data were controlled for eye movement artifacts, and noticeable periods were rejected (threshold 40 μV sd/200 ms moving window). Only artifact-free trials for which the recognition task had been answered correctly entered averaging and ensuing statistical analysis (see below). ERPs were analyzed for four lateral and two midline regions of interest (ROIs) collapsing the data over three electrodes each (left-anterior: F3, FC1, FC5; right-anterior: F4, FC2, FC6; left-posterior: CP5, CP1, P3; right-posterior: CP6, CP2, P4; midline-anterior: Fz, FCz, Cz; midline-posterior: CPz, Pz, POz).

Event-related potentials were calculated as mean voltages. Single-subject averages per condition were calculated by collapsing single trials from 200 ms before to 1000 ms post onset relative to the respective phrase or word onset. Each participant contributed at least 70% of trials per condition and constituent to ERP averaging. The percentage of ERP trial rejections based on false responses and artifact detection was lower than 9% for each critical constituent and in each condition. Logistics regression models on each sentence position revealed that there was no condition-dependent effect on the number of rejected trials (all *p*s > 0.384, see Supplementary Materials for descriptives and analysis).

As all of our experimental items use exactly identical stimulus structure (i.e., identical sequence of word categories), each difference in baseline periods, except for the initial noun phrase, should be due to the critical manipulations and not to differences in the word category of the stimuli. Therefore, i.e., to avoid baseline-induced artifacts in the ERP data, they were not baseline corrected (Drury and Steinhauer, 2012).

Event-related potentials responses were analyzed as mean amplitude voltages for different time windows chosen by visual inspection, focusing on diverging ERP waveforms within the hypothesized N400 time window (noun phrase: 300 – 500 ms; adverbial phrase: 320 – 420 ms; past participle: 430 – 530 ms; see Supplementary Materials for additional analyses supporting the choice of time windows). Grand averages were obtained by collapsing the single-subject averages across participants. Mean amplitude voltages per time window were readout from single-subject average data and analyzed using the hierarchical repeated-measures Analysis of Variance (ANOVA) technique including the fixed factors ANIMACY, TELICITY, AUXILIARY, ROI (region of interest), and the random factor SUBJECT. To avoid excessive Type-I errors due to sphericity violations (*dF* > 1), *p*-values were adjusted using the Huynh and Feldt-correction (Huynh and Feldt, 1970).

Behavioral data of acceptability judgments (again including only trials with correct responses to the word recognition task) were also analyzed with a hierarchical repeated-measures ANOVA including the fixed factors ANIMACY, TELICITY, AUXILIARY and the random factors SUBJECT (*F_1_*) and ITEM (*F_2_*). Responses to the recognition task were only used to control for attentiveness and were not analyzed statistically.

### Results

Data of 28 participants entered the final analysis. Data of one further participant were excluded because of conspicuous behavior during data recording (i.e., requiring substantially more and longer pauses due to circulatory problems).

On average, performance in the recognition task was at ceiling with participants scoring higher than 93% correct responses across critical conditions (see **Table 2**, right column).

**Table 2:** Mean of YES-responses (= acceptable) and correct answers to the recognition task in percent from 28 participants (standard deviations in parentheses).

Condition	Mean of YES-responses (= acceptable) in % (*SD*)	Mean correctness of recognition task in % (*SD*)
Animate-telic (goal), *sein*	94.5 (10.0)	95.9 (4.9)
Animate-telic (goal), *haben*	01.0 (02.4)	93.5 (7.0)
Animate-atelic (loc), *sein*	85.2 (16.2)	95.6 (5.4)
Animate-atelic (loc), *haben*	29.2 (19.9)	95.2 (5.6)
Inanimate-telic (goal), *sein*	90.0 (13.7)	94.7 (6.8)
Inanimate-telic (goal), *haben*	01.7 (04.3)	95.2 (5.0)
Inanimate-atelic (loc), *sein*	76.6 (19.2)	96.8 (5.2)
Inanimate-atelic (loc) – *haben*	32.1 (20.7)	95.8 (5.8)

*Abbr.: loc = locative*.

#### Behavioral Data (Acceptability Judgments)

Acceptability ratings exhibited the following pattern: The factor AUXILIARY has the highest influence aside from a weaker impact of ANIMACY and TELICITY. Items with the auxiliary *sein* were rated as being acceptable much more often than items with *haben.* In addition, ANIMACY and TELICITY seem to modulate the impact of AUXILIARY. Inanimate subjects were less acceptable than animate ones with the auxiliary *sein*. For TELICITY, telic events were rated better with *sein* than with *haben* in contrast to atelic events where items with *haben* received the highest ratings. A repeated-measures ANOVA confirmed this description: Acceptability ratings show main effects of ANIMACY [*F_1_*(1,27) = 7.83; *p* < 0.01; *F_2_*(1,59) = 3.28; *p* < 0.08, marginal for *F_2_*], TELICITY [*F_1_*(1,27) = 18.9; *p* < 0.001; *F_2_*(1,59) = 34.68; *p* < 0.001] and AUXILIARY [*F_1_*(1,27) = 609.93; *p* < 0.001; *F_2_*(1,59) = 1135.83; *p* < 0.001] and the two interactions ANIMACY by AUXILIARY [*F_1_*(1,27) = 12.12; *p* < 0.05; *F_2_*(1,59) = 10.84; *p* < 0.05] and TELICITY by AUXILIARY [*F_1_*(1,27) = 96.18; *p* < 0.001; *F_2_*(1,59) = 129.67; *p* < 0.001]. The two interactions were resolved according to the factor AUXILIARY yielding an effect of ANIMACY only for the auxiliary *sein* [*F_1_*(1,27) = 17.95; *p* < 0.001; *F_2_*(1,59) = 11.02; *p* < 0.01] and an effect of TELICITY for *haben* [*F_1_*(1,27) = 68.07; *p* < 0.001; *F_2_*(1,59) = 107.6; *p* < 0.001] and for *sein* [*F_1_*(1,27) = 28.64; *p* < 0.001; *F_2_*(1,59) = 38.53; *p* < 0.001].

Let us discuss the results of the acceptability task. As assumed (see Stimulus Structure), the factor AUXILIARY has the highest influence on acceptability. See, for instance, the low proportion of YES = acceptable responses for the telic clauses with *haben* vis-à-vis telic clauses with *sein*. This confirms our expectation that the manipulation of the auxiliary draws participants’ task-related attention away from the critical material in the ERP experiment, i.e., subject noun phrase, adverbial phrase and verb lexeme. ANIMACY and TELICITY show a weaker impact on overall acceptability ratings. The reason is that the verbs in our items show no strong preference asymmetries in this regard.

These findings are in line with corpus data. We conducted a pilot corpus study using the Mannheim German Reference Corpus (DeReKo, Institut für Deutsche Sprache, 2012) for the six verbs used in our ERP experiment. Our pilot study confirmed the sharp contrast between *haben* and *sein*. *Haben* is attested for each verb, but it is used more rarely (10.10% of attestations across all six verbs) and it only occurs in atelic contexts. *Sein* is selected much more frequently than *haben* (89.90% across all six verbs) and it is used for each verb in both telic and atelic contexts. By contrast, animacy and telicity asymmetries are less pronounced. We have found 56.79% animate subjects (against 43.21% inanimate ones) and 60.95% telic uses (against 39.95% atelic ones).

#### ERP Data

##### Noun phrase

**FIGURE 1:**
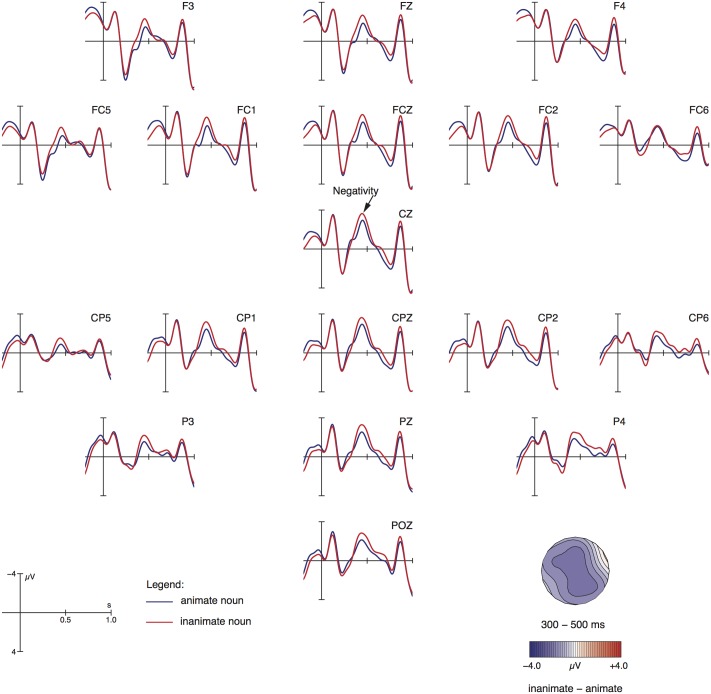
Grand average ERPs relative to the initial noun phrase (onset at the vertical bar) for all electrodes that entered the statistical analysis. Negativity is plotted upward. The topographical map visualizes the negativity effect based on difference waves (inanimate minus animate).

Grand average ERPs relative to the noun phrase are shown in **Figure 1**. As is apparent from **Figure 1**, ERP responses differ for inanimate vs. animate entities between 300 and 500 ms post presentation onset of the noun phrase by showing an N400 for the inanimate conditions against the animate ones. This impression was confirmed by the statistical analysis, which yielded a significant main effect of ANIMACY for midline [*F*(1,27) = 11.13; *p* < 0.01] and lateral electrodes [*F*(1,27) = 12.85; *p* < 0.01] as well as an interaction ANIMACY by ROI [*F*(3,81) = 5.85; *p* < 0.01]. Resolving the interaction by ROI resulted in an animacy effect for left-anterior [*F*(1,27) = 23.22; *p* < 0.001], left-posterior [*F*(1,27) = 11.46; *p* < 0.01] and right-posterior [*F*(1,27) = 8.62; *p* < 0.01] electrode sites but not for right-anterior sites [*F*(1,27) = 2.95; *p* > 0.09].

##### Adverbial phrase

**FIGURE 2:**
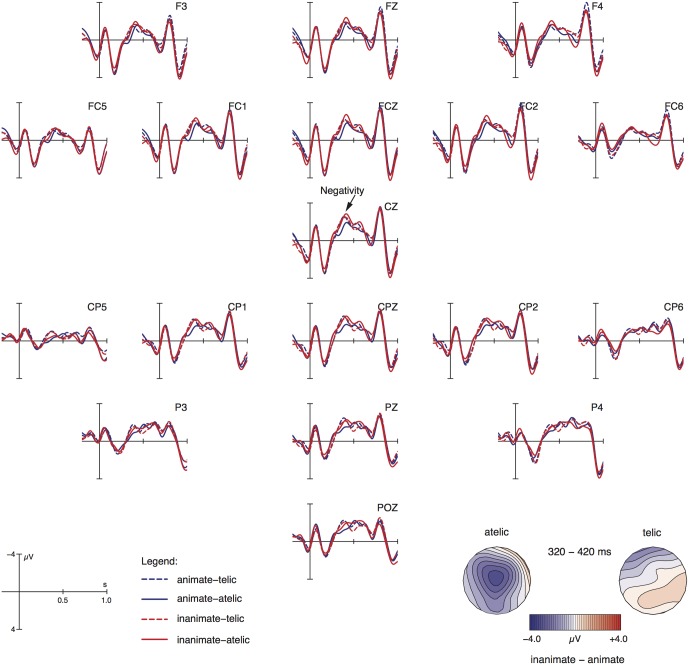
Grand average ERPs relative to the adverbial phrase (onset at the vertical bar) for all electrodes that entered the statistical analysis. Negativity is plotted upward. The topographical maps visualize the negativity effect based on difference waves (inanimate minus animate for telic and atelic conditions).

Grand-average ERPs relative to the adverbial phrase are shown in **Figure 2**. Visual inspection suggests that the most conspicuous differences are visible between 320 and 420 ms post presentation onset of the adverbial phrase. Within this time range, three conditions (animate-telic, inanimate-telic, inanimate-atelic) seem to cluster against the animate-atelic condition by showing a centro-parietally distributed negativity.

Statistical analysis on mean amplitudes confirmed this impression. There was a significant interaction ANIMACY by TELICITY at midline sites [*F*(1,27) = 6.08; *p* < 0.05], being marginally significant for lateral electrodes [*F*(1,27) = 3.36; *p* < 0.08]. Resolving this interaction along the factor TELICITY resulted in a significant animacy effect for an atelic event structure [midline: *F*(1,27) = 9.77; *p* < 0.01; lateral: *F*(1,27) = 4.85; *p* < 0.05], but not for telic events (both midline and lateral electrodes: *F* < 1). In addition, there was a marginal main effect of TELICITY for both midline [*F*(1,27) = 3.90; *p* < 0.06] and parietal electrodes [*F*(1,27) = 3.75; *p* < 0.07].

##### Verbal participle

**FIGURE 3:**
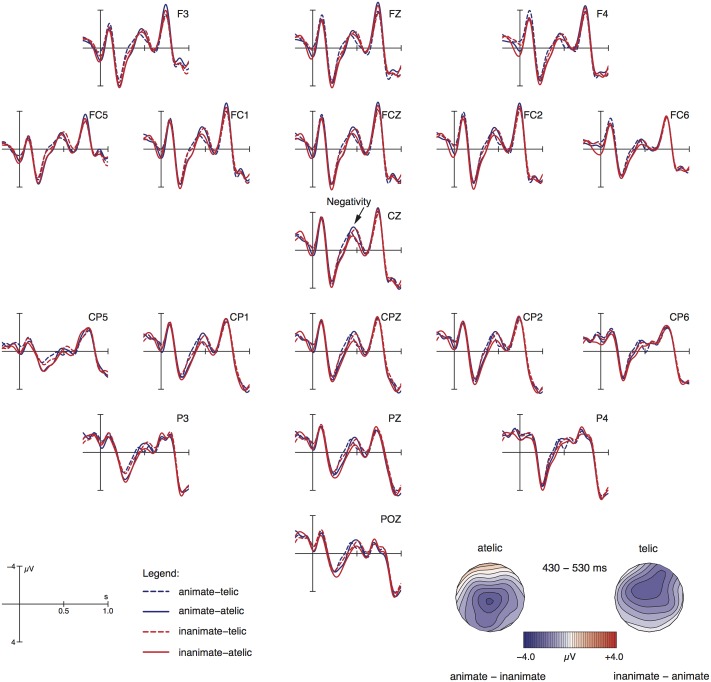
Grand average ERPs relative to the participial verb lexeme (onset at the vertical bar) for all electrodes that entered the statistical analysis. Negativity is plotted upward. The topographical maps visualize the negativity effect based on difference waves (inanimate minus animate for telic conditions and animate minus inanimate for atelic conditions).

Visual inspection of the ERP waveforms relative to the verbal participle (cf. **Figure 3**) revealed that the four conditions differ most obviously between 430 to 530 ms post word onset: both telic conditions (animate-telic, inanimate-telic) and both atelic conditions (animate-atelic, inanimate-atelic) show an animacy effect in terms of a negativity with a maximum at central and fronto-central electrode sites. In particular, in the telic conditions inanimates seem to exhibit an effect against animates, while in the atelic conditions animates seem to evoke a more pronounced negativity compared to inanimates.

Statistical analyses confirmed this description. While no significant main effect could be observed, the interaction ANIMACY by TELICITY was significant for both midline [*F*(1,27) = 14.65; *p* < 0.001] and lateral electrodes [*F*(1,27) = 13.46; *p* < 0.01]. Resolving this interaction along the factor TELICITY showed that the effect of ANIMACY was significant for telic events across all electrode sites [midline: *F*(1,27) = 4.74; *p* < 0.05; lateral: *F*(1,27) = 5.57; *p* < 0.05], while reaching significance in atelic conditions only for midline sites [*F*(1,27) = 4.87; *p* < 0.05]. Lateral electrodes showed a marginal animacy contrast in atelic events [*F*(1,27) = 3.62; *p* < 0.07]. The main effects did not reach significance in this time window.

In addition, visual inspection suggested that, unexpectedly, the ERP waveforms also diverged in a time window preceding the late negativity described above. Specifically, between 230 and 380 ms post word-onset, telic events elicited less positive-going waveforms than atelic ones. Accordingly, statistical analyses for this time window revealed a main effect of TELICITY [midline: *F*(1,27) = 15.42, *p* < 0.001; lateral: *F*(1,27) = 19.10, *p* < 0.001]. Importantly, the interaction with ANIMACY was not significant (all *F*s < 1).

### Discussion

The current experiment revealed that each critical sentence position was associated with particular ERP effects reflecting the incremental processing of animacy and event structure information. At the initial noun phrase, only animacy information was available, while the subsequent adverbial phrase and the participial verb lexeme showed evidence of additional event structure processing. In each case, these effects were manifest as negativities peaking around 400 ms post phrase or word onset. Due to their negative polarity, latency and their topographic distribution, we classified these ERP effects as an N400, well aware of the fact that the N400 is not uniform, but rather a cluster of similar effects (e.g., Kutas and Federmeier, 2011). In the following, we will discuss these findings in light of our predictions for each sentence position.

### Noun Phrase: Processing Animacy

As expected from our prediction (3b), the initial noun phrase referring to an inanimate entity showed increased processing costs in form of an N400 vis-à-vis an initial noun phrase referring to a person. This result is in line with findings from transitive clauses in English (Weckerly and Kutas, 1999) and Tamil (Muralikrishnan, 2011), as described in the introduction.

Given the results obtained at the adverbial phrase, which are best explained by the fact that agent assignment has not been completed before the verb in our study, we propose a minimality-based explanation in terms of semantic roles and assume that sentence-initial animate subjects are not assigned a specific semantic role before the verb (cf. Weckerly and Kutas, 1999; Paczynski and Kuperberg, 2009). Animate subjects may cover a wide array of roles from volitional agents over experiencers or self-propelled movers to patient-like roles (cp. *The paraglider worked / felt cold / was hit*). Hence, they are less restricted in terms of semantic roles and verb types compared to inanimate subjects. By contrast, it is more difficult or impossible to interpret inanimates as volitional agents, experiencers or self-propelled movers. This means that when encountering an animate subject, the processing system may keep role specification, i.e., assignment of role properties, to a minimum. By contrast, when encountering an inanimate subject, it is confronted with increased processing costs by eliminating the above-mentioned proto-agent properties as an option. This type of explanation can be subsumed under the more general minimality processing principle (e.g., Bornkessel et al., 2004; Hawkins, 2004; Bornkessel-Schlesewsky and Schlesewsky, 2009b): During online comprehension, semantic (and syntactic) specifications are kept to a minimum until more information is present either in the utterance itself or the context.

Our results seem puzzling in the light of previous ERP findings from transitive clauses in German and Mandarin Chinese [see Prediction (3a)]. They reveal no differences between inanimate and animate initial arguments (see Bornkessel-Schlesewsky and Schlesewsky, 2009a for an overview for German; Philipp et al., 2008 for Chinese). As mentioned in the introduction, differences between our study and previous work might help understand this discrepancy. The above-mentioned studies tested transitive constructions with two preverbal nominal arguments. As the items of these studies employed a similar constituent order even in filler sentences, it seems reasonable to consider that the item structure itself may have shaped a particular incremental expectation. Due to the transitive stimulus structure, participants may have always expected a second argument thereby being used to interpret animacy as a relational feature between two arguments. This might have postponed the evaluation of animacy from the first noun phrase to the second. In our study, the item structure, which was also uniform across all conditions including fillers, might have played a role as well. The initial noun phrase in our design is expected to be the single nominal argument of the clause so that this is the only nominal position where animacy could be evaluated.

Importantly, Philipp et al. (2008) and Bornkessel-Schlesewsky and Schlesewsky (2009a) assume that animacy of the initial subject does not suffice for agent identification in German. Our results for the initial subject and the adverbial phrase (see below) are fully compatible with this assumption. Although we have found an animacy effect for the subject, the results for the adverbial phrase prompt us to assume that agent identification has not been completed before the verb lexeme in our study.

While it is uncontroversial that animacy effects on sentential subjects are primarily seen on the N400 component, previous research offers various explanations for the processing advantage of animate subjects against inanimate ones (cf. the overview in Paczynski and Kuperberg, 2011). Besides different explanations in terms of semantic roles (see also the introduction of the current paper), frequency of occurrence, which is known to modulate the N400 (e.g., Kutas and Federmeier, 2011), is another possible explanans. Animate subjects are more frequent than inanimates ones, so this might be an explanation for their processing advantage. Indeed, as shown by Fischer (2013) in a contrastive corpus study of German and English, this frequency distribution holds for transitive clauses in both languages. However, as mentioned above, the N400 effects observed for inanimate vs. animate subjects of transitive clauses in German and English differ – a cross-linguistic processing difference that cannot straightforwardly be accounted for by frequency statistics. Therefore, we prefer an explanation of our results in terms of semantic roles being aware that this explanation for animacy effects is still an open question which has to be addressed by further studies.

### Adverbial Phrase: Processing Event Structure

At the position of the adverbial, locative vs. goal, phrase we observed an animacy difference in the atelic conditions, i.e., the inanimate-atelic condition showed an N400 effect in comparison to the animate-atelic condition. The two telic conditions did not differ in terms of animacy; our visual impression is that these two conditions exhibit an amplitude similar to the inanimate-atelic condition.

Our results are in line with prediction (5) and the general processing assumption that the processing system prefers minimal syntactic and semantic specifications during online comprehension (e.g., Bornkessel et al., 2004; Hawkins, 2004; Bornkessel-Schlesewsky and Schlesewsky, 2009b). As mentioned in the introduction, a goal phrase indicates a complex event with a change of location. In addition, goal phrases are verb arguments thereby adding an argument to the argument structure of the clause. By contrast, a locative phrase does not increase the complexity of the event and argument structure. The locative phrase functions semantically as a modifier in our critical items. As the critical verbs are indeterminate regarding telicity, the event structure of an item with a locative modifier is atelic, i.e., lacks the component of change of location. Hence, atelic situations, i.e., conditions with a locative adverbial in our study, require a smaller number of relevant specifications than telic events, i.e., conditions with goal adverbials. Second, noun phrases that restrict the range of expected role interpretations, i.e., inanimate conditions, are more demanding than animate nominal arguments that are compatible with a broader range of possible role interpretations (cf. discussion of the findings for the noun phrase above). Thus, both information types, i.e., inanimate noun phrases and goal phrases, restrict possible interpretations and therefore increase processing complexity.

This leads us to conclude that the simplest condition in terms of minimality is the animate-atelic condition and that each deviation from this increases complexity in terms of interpretation demands. This is indeed the pattern we found in our data at the adverbial phrase: the inanimate-atelic condition showed a centrally distributed negativity against the animate-atelic condition. We interpret this effect as being caused by interpretation restrictions with respect to the nominal argument. The telic conditions did not differ in terms of animacy and were not directly comparable (statistically) to the animate-atelic condition. Therefore, we can only safely conclude that on the adverbial phrase both telic conditions do not differ in terms of interpretive complexity. It is only by visual inspection that we suggest that these two conditions exhibit a similar amplitude like the inanimate-atelic condition.

### Verbal Participle: Integrating Semantic Role and Event Structure Information

The third and last item of interest in our experiment is the verbal participle. We recapitulate our results for ease of exposition. In the telic conditions, which refer to a goal-oriented motion, the inanimate conditions exhibit an N400 effect against the animate conditions. In the atelic conditions, which express an aimless motion, items with animates evoked an N400 effect against inanimate conditions.

At this stage, three types of information are assembled: the event structure inferred from the semantics of the adverbial phrase, the verb meaning referring to a process of motion and animacy information, which together with the verb meaning triggers role identification. The results found prior to the verb participle prompt us to assume that role (agent) identification only takes place when the verb is encountered.

The results on the verbal participle are pertinent to the issue whether change of state (or location) is a patient or an agent property. They call into doubt the opinion in theoretical linguistics mentioned in the introduction (e.g., Dowty, 1991; Zaenen, 1993; Ackerman and Moore, 1999) that a change of location, as in the telic conditions, is a characteristic property of the patient role. If this were true, the animate conditions should have exhibited increased processing costs in the telic conditions since an agent going through a definite change of location is claimed to have conflicting role properties. As an animate entity it is preferentially interpreted as an agent, its definite change of location characterizes a patient in this line of research. Correspondingly, in the atelic conditions the inanimate items should have been harder to process since they lack the supposedly crucial patient property of a definite change of state [see Prediction (6) above].

We found opposite patterns. Our results are consistent with studies on ontogentic and phylogenetic language development claiming that goal-directed behavior characterizes – among other properties such as autonomous movement – agents and animates as agents (Rakison and Poulin-Dubois, 2001; Carpenter et al., 2005; Spelke and Kinzler, 2007; Carey, 2009). In this view, a participant that changes his/her location in a goal-directed way independently of another participant is a more prototypical agent than a participant that moves aimlessly. Our results support the predictions of this line of research [see Prediction (7) above]. The role of an inanimate entity involved in a definite (goal-oriented) change of location (our telic condition) has inconsistent role properties. As an inanimate entity it is preferentially interpreted as a patient, its goal-oriented behavior qualifies it as an agent. Therefore, it is expected to engender increased processing costs vis-à-vis an animate entity involved in a goal-oriented change of location, which is what we have observed. Correspondingly, in the atelic conditions, which express an aimless motion, items with an animate referent were more difficult to process than items with inanimate referents because aimlessly moving animates are less prototypical agents lacking the agent property of goal-directedness.

While our predictions focused on the N400 component, we also observed an unexpected result in the form of an early positivity before the N400 time window. This positivity was reduced for telic events compared with atelic ones. This effect seems similar to the early positivity reported for the beginning of the agent phrase (in e.g., *the actress worshipped/awakened by the writer*) in Malaia et al. (2009), yet differs from it in several ways. Firstly, we registered the effect on the participial verb lexeme, whereas Malaia and colleagues did not find early telicity effects on the verb. Secondly, our positivity also differs in terms of its somewhat longer latency, its broad topographic distribution, and in the fact that Malaia and colleagues report that telic events were more positive than atelic ones. We conclude that telicity effects in early time windows clearly require further systematic investigation.

## General Discussion

The present ERP study investigated the contribution of the verb’s co-constituents to the interpretation of semantic roles and event structure and explored how these pieces of information are assembled step-by-step with respect to each other and with the verb’s meaning. To this end, we focused on verb-final structures in German with verbs of motion such as *fliegen* ‘fly’ and *schweben* ‘float, hover.’ These verbs are indeterminate with respect to agentivity and telicity as they are compatible with animate and inanimate subjects as well as with locative and goal phrases. We have measured ERPs relative to the animate vs. inanimate subject noun phrase, the locative vs. goal phrase and the verbal participle. An additional manipulation was the choice of the auxiliary *sein* ‘be’ vs. *haben* ‘have,’ which showed pronounced acceptability differences and served to draw participants’ task-related attention away from the previously occurring animacy and telicity variation. The main results of our study and the general explanations we have proposed are summarized in **Table 3**.

**Table 3:** Summary of the ERP results and proposed explanations. Conditions that evoked N400 effects are marked bold.

Condition	Noun phrase	Adverbial phrase	Verbal participle
Animate-atelic (loc)	*der Gleitschirmflieger*	*über dem Fluss*	***geschwebt*** **N400**
	the paraglider	above the river	floated
Animate- telic (goal)		***auf den Acker*** **N400**	*geschwebt*
		to the ground	floated
Inanimate-atelic (loc)	***das Ahornblatt*** **N400**	***über dem Fluss*** **N400**	*geschwebt*
	the maple leaf	above the river	floated
Inanimate- telic (goal)		***auf den Acker*** **N400**	***geschwebt*** **N400**
		to the ground	floated

Explanation	Minimal role specification	Minimal role & event structure specification	Role identification and role prototypicality

*Abbr.: loc = locative*.

Regarding the subject noun phrase, inanimates showed increased processing costs in form of an N400 vis-à-vis animates. At the adverbial phrase, we observed an animacy difference in the atelic (locative) conditions: the inanimate-atelic condition showed an N400 effect in comparison to the animate-atelic condition. The two telic conditions did not differ in terms of animacy; our visual impression was that these two conditions exhibit a similar amplitude like the inanimate-atelic condition, as shown in **Figure 2** and summarized in **Table 3**. Finally, at the verbal participle, the inanimate conditions exhibit an N400 effect against the animate conditions in the telic contexts, which refer to a goal-oriented motion. In the atelic conditions, which express an aimless motion, items with animates evoked an N400 effect compared to inanimates.

Our explanations proposed for these findings can be followed more easily if we start with a general discussion of the results for the verbal participle. These results are relevant for our main issue whether change of state (or location) is a patient or an agent property. Our results support the claim that a participant that changes his/her locational state in a goal-directed way independently of another participant exhibits a prototypical agent property (Rakison and Poulin-Dubois, 2001; Carpenter et al., 2005; Spelke and Kinzler, 2007; Carey, 2009). In this view, the role of an inanimate entity involved in a definite (goal-oriented) change of location (our telic condition) has inconsistent role properties. Its goal-oriented behavior qualifies it as an agent, but lack of animacy disqualifies it for this role. Therefore, the inanimate-telic condition was expected to engender and indeed exhibited increased processing costs vis-à-vis the animate-telic condition. Correspondingly, in the atelic condition expressing an aimless motion, animates were more difficult to process than inanimates. The reason is that inanimates are expected to lack goal-directedness, which in this view characterizes animate agents. Our findings cast doubt on the opinion in theoretical linguistics that a change of location, as in the telic condition, is a characteristic property of the patient role (e.g., Dowty, 1991; Zaenen, 1993; Ackerman and Moore, 1999).

A viable way to reconcile the opposed views is to assume that the classification of change of location depends on causation. If the participant is the instigator of his/her own change of location in the event named by the verb, it is an agent property. If the change is caused by another participant, it is a patient property. This type of explanation is proposed by Dowty (1991, p. 574) for movement, which he classifies as a proto-agent property, but only when not caused by another participant in the event named by the verb. For Dowty, causation has priority over movement for distinguishing agents from patients. Our assumption is that this also holds for change of location, which is a specific type of movement, and possibly for change of state in general.

The N400 effects we have found relative to the verb lexeme are in line with and thus support previous literature where this effect is taken to reflect problems with semantic retrieval in general (Kutas and Federmeier, 2011). In particular, this effect has been found for participants with less prototypical roles in structures with two nominal arguments (e.g., Philipp et al., 2008; Bornkessel-Schlesewsky and Schlesewsky, 2009a; Nieuwland et al., 2013). As a novelty, our study investigated role processing in structures with only one nominal argument. In our study, processing disadvantages were also caused by prototype violations, which were of two types. First, difficulties arise when role properties are inconsistent. This happens, for instance, when agent properties that are typical for animates such as moving independently in a goal-directed way are assigned to inanimates. Second, difficulties emerge when roles lack a crucial prototype-defining property, for instance, when agents (animates) move aimlessly. Whether the two types of departures from the role prototype – inconsistent or lack of characteristic role features – can be disentangled is a question for future experimental research.

For the N400 effects found on the subject noun phrase and the adverbial phrase we offered an explanation based on the generally acknowledged processing principle of minimality or simplicity (e.g., Bornkessel et al., 2004; Hawkins, 2004; Bornkessel-Schlesewsky and Schlesewsky, 2009b). According to this principle, semantic and syntactic specifications are kept to a minimum during online comprehension until more information is present either in the utterance itself or the context. Let us start with complexity asymmetries arising at the adverbial phrase. The goal-phrases in our telic conditions add specifications to the sentence in terms of argument structure, since they are syntactic and semantic arguments, and in terms of event structure, by adding a change of location component (Maienborn, 1994; Maienborn and Schäfer, 2011). This means that an additional argument and an additional event structure specification have to be processed. By contrast, locative phrases are non-arguments that do not increase the complexity of the event structure. In conjunction with the complexity asymmetry stemming from the animacy distinction, we expected to observe and indeed found N400 effects in the three conditions – animate-telic, inanimate-telic and inanimate-atelic –, which are more complex compared to the animate-atelic condition.

A comparison between our results and the effects reported in the few previous ERP studies devoted to telicity (e.g., Malaia et al., 2009, 2012; Malaia and Newman, 2015) reveals considerable differences (see Introduction and Verbal Participle: Integrating Semantic Role and Event Structure Information). We explained these differences by the varying types of constructions under analysis.

Now let us turn to the animacy-related complexity of the sentence initial nominal argument. Sentence initial noun phrases that impose restrictions regarding their semantic role, i.e., which are incompatible or highly improbable with certain roles, are semantically more complex than sentence initial noun phrases that impose no such restrictions. Adapting Weckerly and Kutas’ (1999) explanation for the N400 effect evoked by inanimate subjects in English, we assume that inanimates impose such restrictions since they are incompatible or highly improbable with many agentive properties. In processing terms, when encountering a sentence initial inanimate subject the processing system eliminates agentive specifications as an option. By contrast, animate subjects are compatible with virtually all semantic roles and hence the processing of their putative semantic role is less demanding: the human processor does not need to make any role specifications at all. This kind of explanation seems to defy incrementality. However, immediate analysis does not necessarily mean that linguistic information is always interpreted to the fullest degree possible (Nieuwland and Van Berkum, 2005).

In our view, semantic role identification, i.e., a full-fledged role interpretation, has not been completed before the verb in our experimental set up. This explains why the presence or absence of the change of location meaning component provided by the adverbial phrase did not evoke role prototypicality effects. Information in terms of animacy and change of location was not sufficient for role identification and role prototypicality effects, which only occurred when the verbal lexeme was processed, as described above. Our analysis is in line with the assumption of Bornkessel-Schlesewsky and Schlesewsky (2009a) that animacy by itself is insufficient for agent assignment in German. So, despite the fact that they report no animacy effects on initial subject noun phrases in German transitive clauses while we found such effects in clauses with a single nominal argument, we share the observation that animate initial arguments are not interpreted as agents in the absence of additional information. Agent identification is completed when the verb lexeme is encountered, as observed in our experiment, or at the second argument when two arguments in the clause are interpreted relative to each other, as shown in the studies reported by Bornkessel-Schlesewsky and Schlesewsky (2009a).

As we have proposed in the introduction, animacy effects in sentence processing may be dependent, inter alia, on experimental setup with respect to stimulus structure and choice of fillers. In our study, the use of intransitive structures throughout critical and filler items may have shaped participants’ predictions about the upcoming clause structure so that animacy evaluation took place at the first nominal argument in the clause. By contrast, in prior studies using transitive structures (e.g., Roehm et al., 2004; Philipp et al., 2008) animacy evaluation took place at the second nominal, because participants anticipated a two-argument structure. One could argue that our findings might not be robust enough because the critical sentences were not embedded in a more natural context in which further information cues may interact with argument predictions. There is, however, evidence that animacy effects in the N400 are replicable when ERPs are collected in rich naturalistic contexts (e.g., Alday et al., 2016, unpublished)^4^. Nevertheless, further research and replication is clearly needed for our novel finding of an animacy-by-telicity interaction at the adverbial phrase and the verbal participle.

Despite these considerations, the finding of N400 effects for inanimate sentential subjects is uncontroversial, even though the explanations offered in previous research for the processing advantage of animate subjects against inanimate ones vary considerably (cf. the overview in Paczynski and Kuperberg, 2011). Some explanations assume that animate subjects show a processing advantage over inanimate ones since they are prototypical agents (e.g., Muralikrishnan, 2011). This presupposes early agent identification. However, our results are not compatible with the latter assumption. Another possible explanation is based on the observation that animate subjects are more frequent than inanimates ones and frequency of occurrence is known to modulate the N400 (e.g., Kutas and Federmeier, 2011). As mentioned in Section Noun Phrase: Processing Animacy above, frequency and ERP data are in conflict with each other in transitive clauses in English and German. This reduces the appeal of this type of explanation in comparison to our minimality-driven account. Considering these divergent views, processing of animacy in German and other languages still needs further experimental investigation.

## Author Contributions

MP designed the experimental task and conducted the experiment. TG and BP designed the experimental task and wrote the article. MP, TG, and FK ran the statistical analysis and wrote the article.

## Conflict of Interest Statement

The authors declare that the research was conducted in the absence of any commercial or financial relationships that could be construed as a potential conflict of interest.

## Footnotes

1 https://dgfs.de/de/inhalt/ueber/ethikkomission.html

2 www.dfg.de/foerderung/faq/geistes_sozialwissenschaften/

3 http://wortschatz.uni-leipzig.de/

4 https://doi.org/10.1101/062299
