# Supplementary material for: Beyond Verb Meaning: Experimental Evidence for Incremental Processing of Semantic Roles and Event Structure
Source: Front Psychol. 2017 Oct 30;8:1806. doi: 10.3389/fpsyg.2017.01806 (PMC5670351; doi:10.3389/fpsyg.2017.01806)
Supplement: Supplementary file 2 [file Data_Sheet_2.PDF]

Supplementary Material

Beyond verb meaning: experimental evidence for incremental processing of semantic roles and event structure

Markus Philipp*, Tim Graf, Franziska Kretzschmar, Beatrice Primus

*** Correspondence:** Markus Philipp: markus.philipp@uni-koeln.de

1 Supplementary Data Analysis

1.1 Sliding Window Analysis

To provide another method to corroborate our choice of analyzed time windows, we applied a repeated-measures ANOVA on the single-subject mean amplitude voltages in consecutive 20 ms time windows starting from word or phrase presentation onset. The specifications of each ANOVA were identical to the ones reported in section 2.3.2 in the main text. The number of factors are dependent on the sentence position. While at the noun phrase only the factors ANIMACY and ROI are relevant, the factor TELICITY becomes relevant for the two other positions. We included all possible interactions. For the assessment of significance, we used the Huynh and Feldt-corrected *p*-values, whenever sphericity violations are present. For reasons of clarity of the presentation we did not resolve significant interactions (see main text for resolved interactions). As main effects of ROI are not informative for our investigation, these are not depicted below.

1.1.1 Results of the Supplementary Data Analysis

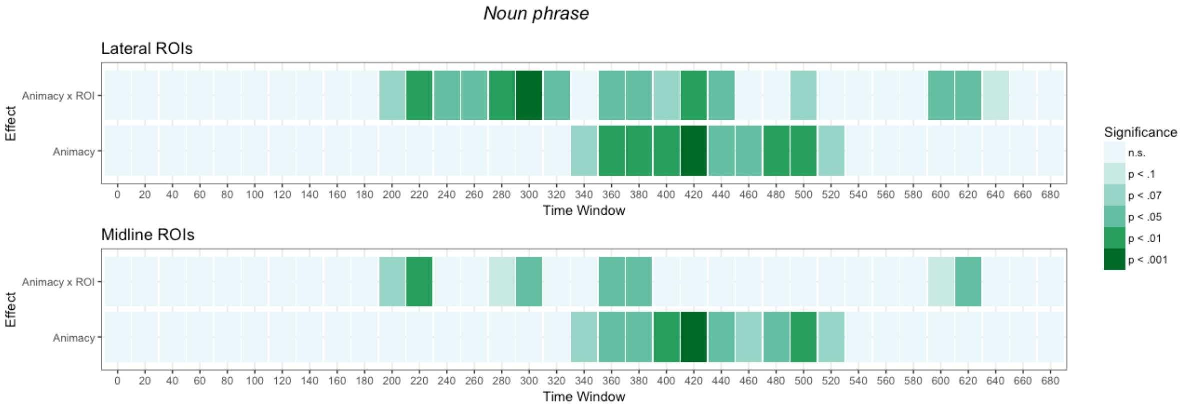

Supplementary Figure 1. Results of a consecutive 20ms time window ANOVA analysis at the position of the noun phrase. Values on the x-axis represent the beginning of a time window. The y-axis depicts the factors with decreasing complexity from top to bottom. Colors represent the *p*-value, with darker colors indicating a smaller *p*-value.

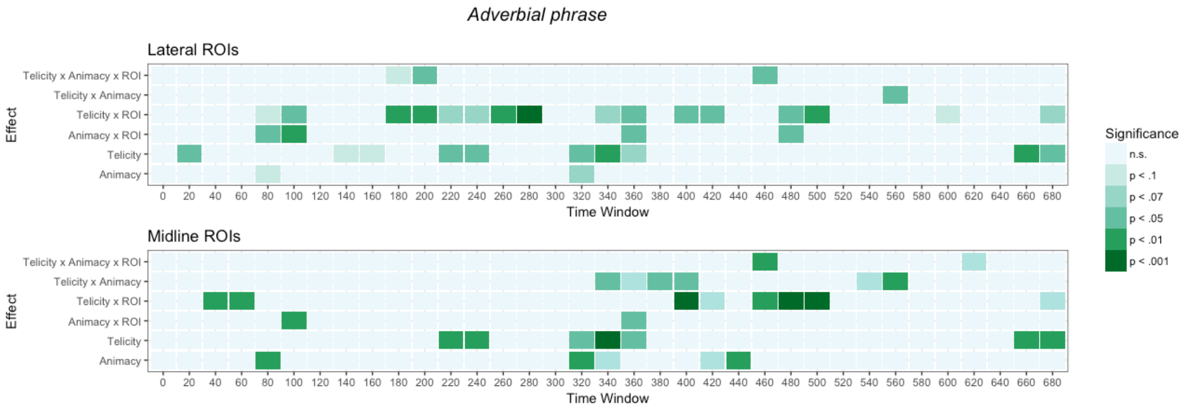

Supplementary Figure 2. Results of a consecutive 20ms time window ANOVA analysis at the position of the adverbial phrase. Values on the x-axis represent the beginning of a time window. The y-axis depicts the factors with decreasing complexity from top to bottom. Colors represent the *p*-value, with darker colors indicating a smaller *p*-value.

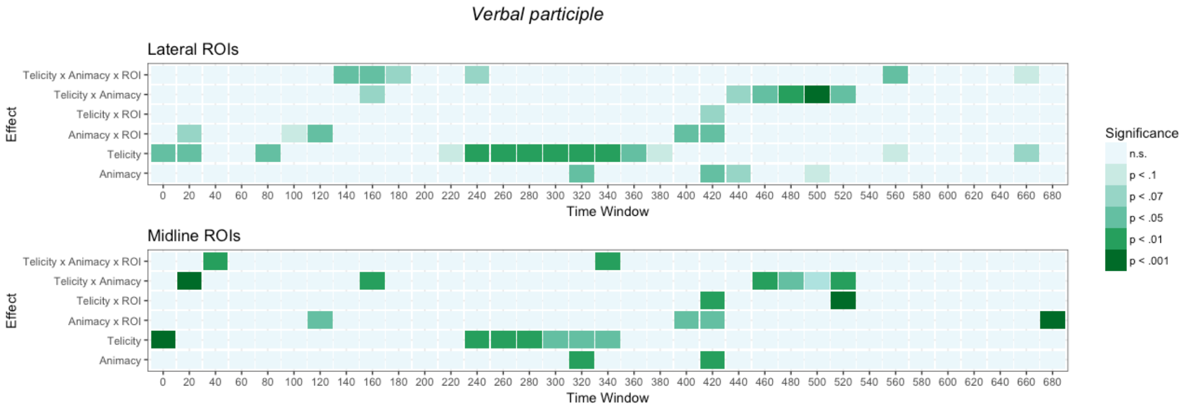

Supplementary Figure 3. Results of a consecutive 20ms time window ANOVA analysis at the position of the verbal participle. Values on the x-axis represent the beginning of a time window. The y-axis depicts the factors with decreasing complexity from top to bottom. Colors represent the *p*-value, with darker colors indicating a smaller *p*-value.

1.2 Rejection statistics

1.2.1 Descriptives

We provide the number of included and rejected trials, as well as the percentage of included trials, per condition for all three sentence positions.

Noun Phrase

Condition	Included trials	Rejected trials	Percentage of trials included
animate	1554	126	92.5
inanimate	1557	123	92.7

Adverbial phrase

Condition	Included trials	Rejected trials	Percentage of trials included
animate telic	780	60	92.9
animate atelic	787	53	93.7
inanimate telic	788	52	93.8
inanimate atelic	803	37	95.6

Verbal participle

Condition	Included trials	Rejected trials	Percentage of trials included
animate telic	765	75	91.1
animate atelic	774	66	92.1
inanimate telic	783	57	93.2
inanimate atelic	787	53	93.7

1.2.2 Inferential statistics

In order to assess whether there are condition-dependent differences in the number of rejected trials, we fitted logistic regression models on the proportions of included and rejected trials for each sentence position. At the noun phrase we included the preditor ANIMACY; at the adverbial and the verbal participle we included ANIMACY, TELICITY and their interaction as predictors. We used Wald's z-test on the regression coefficients to determine statistical significance. We report the coefficient table. Note that the effect of intercept is irrelevant because it only indicates that the reference level is not equal to chance level.

Noun phrase

	**Estimate**	**Std. Error**	***z*-value**	***p*-value**
Intercept	2.525	0.066	38.341	< .001
ANIMACY	-0.013	0.066	-0.198	.843

Adverbial phrase

	**Estimate**	**Std. Error**	***z*-value**	***p*-value**
Intercept	2.505	0.691	3.623	< .001
ANIMACY	-0.073	0.450	-0.162	.871
TELICITY	-0.093	0.448	-0.208	.835
ANIMACY x TELICITY	0.226	0.295	0.767	.442

Verbal participle

	**Estimate**	**Std. Error**	***z*-value**	***p*-value**
Intercept	1.823	0.629	2.900	.003
ANIMACY	0.359	0.413	0.871	.384
TELICITY	0.201	0.404	0.498	.619
ANIMACY x TELICITY	-0.062	0.265	-0.233	.815
