# Supplementary material for: Beyond Verb Meaning: Experimental Evidence for Incremental Processing of Semantic Roles and Event Structure
Source: Front Psychol. 2017 Oct 30;8:1806. doi: 10.3389/fpsyg.2017.01806 (PMC5670351; doi:10.3389/fpsyg.2017.01806)
Supplement: Supplementary file 3 [file Image_1.PDF]

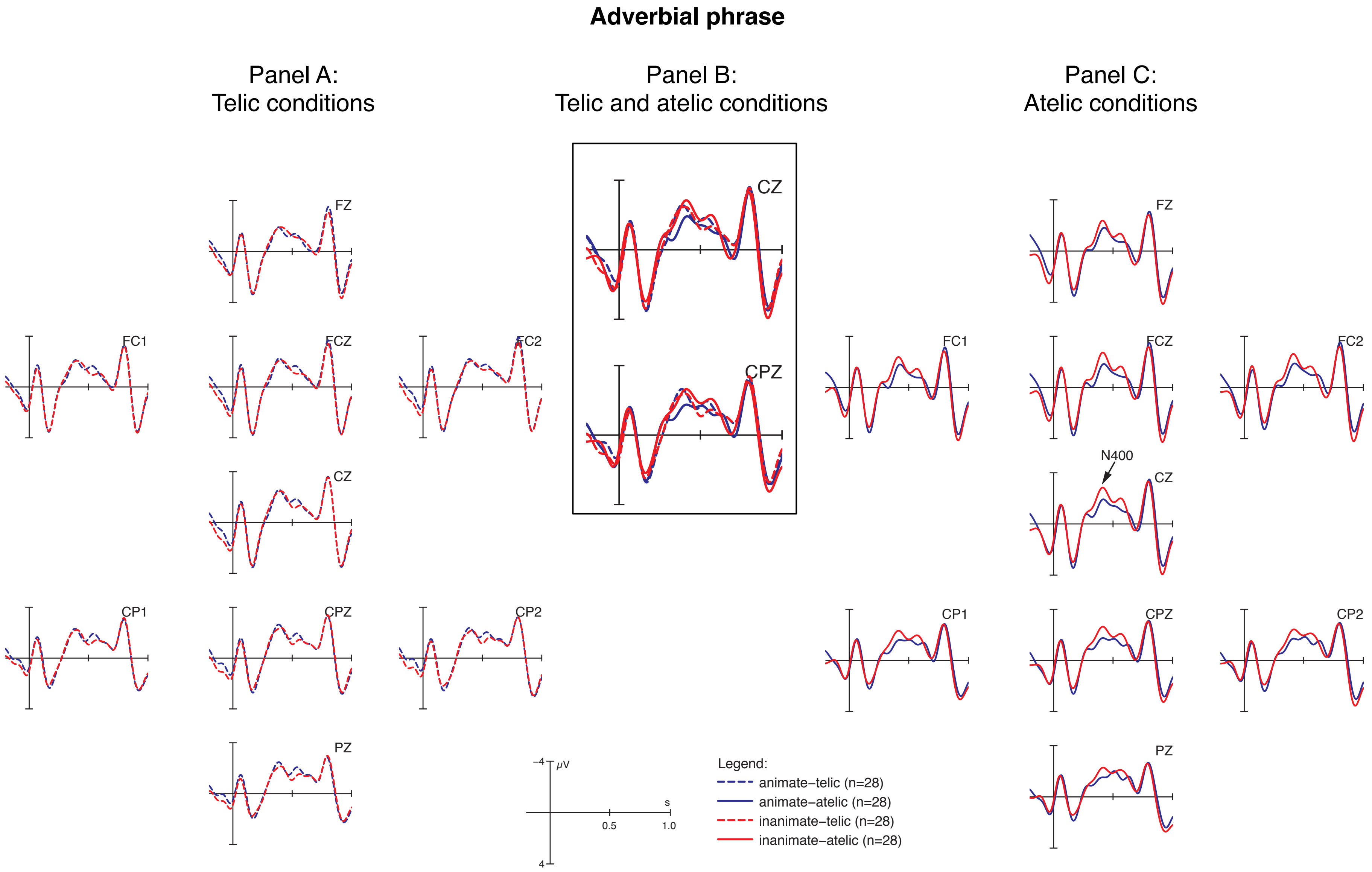

Grand average ERPs relative to the adverbial phrase (onset at the vertical bar) for a subset of electrodes. Conditions were separated by TELICITY. Panel A: Telic conditions. Panel C: Atelic conditions. Panel B shows all conditions at Cz and CPz. Negativity is plotted upwards.
